# Book Review

**Published:** 1926

**Authors:** 


### Reviews of Books.

**What it Feels Like.** By "DOCTOR ROBIN." Pp. 78. Paper cover. London: Student Christian Movement. Price 2s.—It is often indiscreet to keep old letters, but fortunately those of "Dr. Robin" to his old friend Bill were not destroyed. "Dr. Robin" is a teacher in a large Chinese school of medicine, and in *What it feels like—Letters from a Doctor out East to a Colleague at Home*, he makes one interested in, sympathetic for, and even envious of his job. There are most thrilling stories of his work, telling of tropical diseases and of famine, of student life in China and of casualty work with the army of the so-called Christian General Feng-Yu Hsiang. He shows that a missionary can be—and that a real one is essentially—a normal man. This book provides most entertaining reading and much food for thought when it is finished.

**Gould's Medical Dictionary.** Edited by R. J. E. SCOTT, M.D. Imperial octavo. Pp. xi., 1,398. Illustrated throughout. Maple Press Co., York, Pa., U.S.A. London: H. K. Lewis & Co. Ltd. 1926. 30s. net.—That since 1890 no less than 700,000 copies of this standard work have been sold is sufficient evidence of a continuing demand. The present edition claims 1,400 pages, and 76,000 words, of which 5,000 are new. As the editor felicitously explains, "The skilful plan, wide scope, and distinctive qualities of the previous lexicons have been maintained." Even such humble words as "quiz" and "turps" have their place. The unhappy practice of naming discoveries and processes from those who describe them makes a work of this kind invaluable to the general reader. Here he will find Adam's pill, Baelz's disease, Farr's tubercle, Garel's sign, Narath's operation and Penzoldt's test. A host of proprietary names for well-known drugs forms no small part of the book. Those who have never mastered the new nomenclatures will welcome the anatomical tables, while other exhaustive lists, such as that of bacteria, increase the value of the work for reference. The seeker after information whom this book does not satisfy must be hard to please. One or two definitions are unusual, *e.g.* "nyctalopia, night vision . . . the sight is better by night," instead of the opposite and customary meaning. The statement that contramine is the "same as intramine" is inaccurate. The spelling is unreliable. Such forms as "tumor, color" one must expect and condone, but there is no need for "cumin." On the vexed question of the final "e" the editor prefers a definite system agreement with common practice; where he departs from it, it is often hard to know why, *e.g.* "carmin, margarin, phosphatid, alypin" (but "xanthine, lipide, stovaine"). "Adrenalin" is used throughout the text, but "adrenaline" is the only spelling given. More seriously objectionable is the treatment of words of classical origin. "Esophagus, cecal, hematoma" are an offence, and this practice tends to avoidable confusion: "edemania" (aedoemania) and "edema" (oedema), "celology" and "celom" (coelom), "amenomania" (amaenomania) and "amenia" are nowise related. The method is not even consistently maintained: "allesthesia, see allocheiria," "allocheiria, see allochiria"; "cherophobia" is preferred, but "chaeromania," "esthematology, aesthema, enomania, oenilism, celiemia, coelioplegia" all appear without alternative. The text is clear, the paper good, and the whole volume extremely well got up.

**A Guide to Anatomy: for Students of Medical Gymnastics, Massage and Medical Electricity.** By E. D. EWART. Second Edition. 8vo. Pp. xiv., 336. 4 coloured plates, 93 illustrations. London: H. K. Lewis & Co. Ltd. 1926. Price 12s. 6d.—This volume represents the second edition of a manual designed for the instruction of massage pupils in anatomy, and deals fully and clearly with those parts of the subject which are more especially in the province of the masseuse, and more sketchily with the remainder of it. In looking through the work, one is impressed once more with the vast amount of anatomical knowledge which is required of the pupil, and also with the fact that here she will surely find all the knowledge of the theoretical side of her subject set forth in a systematic and helpful way. There are a few minor details to which attention might be directed in the preparation of any future edition. The statement that "it (the small intestine) is suspended to the spinal column by the mesentery" is not good either as grammar or anatomy, while it is open to doubt whether the spinal accessory nerve can be recognised by palpation at the posterior border of the sterno-mastoid muscle. It would also be well in a book on a subject which pupils find so difficult to decide whether to talk of the pelvic colon or the sigmoid flexure, and to be consistent in the use of the term selected. The book is as clear and readable as such a text-book can be, and we recommend it with confidence to those for whom it has been written. Features worthy of commendation are the use of the "old" terminology, and the illustrations, which are clear and admirably adapted to their purpose.

**Reports of the St. Andrew's (James Mackenzie) Institute for Clinical Research, St. Andrew's, Fife.** Vol. III. Edited by Professor David Waterston, M.D., F.R.C.S. (Edin.). Pp. 227. Oxford Medical Publications. 1926. Price 10s. 6d.—This new volume of the St. Andrew's Reports opens with a reproduction of Gordon Shield's portrait of Sir James Mackenzie. Like most of Mackenzie's portraits, it gives the impression of an austere and rather forbidding personality. Nothing could be further from the truth, as we find in Maitland Ramsay's address on Mackenzie which comes first in the printed matter of this volume. Several of the other papers are of considerable interest. Dr. Alexander Hynd found that among 14 cases of eclampsia there were two groups: in one the optical activity of the urinary albumin was that of serum albumin, in the other it was that of lactalbumin. He suggests, therefore, that in some cases eclampsia is an anaphylactic reaction to foreign protein escaping into the circulation from the mammary gland. We trust this discovery will not revive the barbarous practice of breast amputation as a treatment of eclampsia, which had a brief vogue in certain continental clinics some years ago. In another interesting paper Dr. James Orr has assembled a number of cases of intermittent limp and allied phenomena. Dr. J. H. P. Paton gives a valuable analysis of his notes on a large number of school-girls, one of his points being that the size of the tonsil is not a satisfactory index of its morbidity.

**The Principles of Diagnosis and Treatment in Heart Affections.** By Sir James Mackenzie, M.D., F.R.S., etc., and James Orr, M.B., Ch.B. Third edition. Pp. viii., 242. Oxford Medical Publications. 1926. Price 10s. 6d.—It is not surprising that this book should have reached a third edition, for it embodies in a compact and easily readable form Mackenzie's principles of cardiology in their application to every-day practice. Dr. Orr has succeeded in imparting the flavour of the St. Andrew's work to the book without increasing its bulk. It is fortunate that an exposition of Mackenzie's teaching, including its last phase, is available in so handy a form ; and although it is unlikely that any further edition will be called for, this one will doubtless enjoy a long period of popularity.

**The Heart.** By A. G. GIBSON, D.M., F.R.C.P. London. Pp. vii., 108. Oxford Medical Publications. 1926. Price 5s. —This is one of the new " Oxford Medical Handbooks " which, we are told in a publisher's note, are not short text-books, but only brief surveys of general principles. Nevertheless, Dr. Gibson has succeeded in compressing a marvellous amount of information into the very small space allotted to him. The book can be read through in a few hours, and students as well as practitioners may be trusted to derive from it a well-balanced conception of modern cardiology.

**Modern Methods in Heart Disease.** By FRANCIS HEATHERLEY, M.B., B.S. (Lond.), F.R.C.S. Second edition. Pp. xii., 269. London: Baillière, Tindall & Cox. 1926. Price 8s. 6d.—To the first edition, which we reviewed in 1923, Dr. Heatherley has now added a chapter on blood-pressure ; and, in an appendix, some hints on the home treatment of functional disorders of the heart. These hints are also printed separately in pamphlet form, for distribution to patients. Dr. Heatherley writes in a cheerful, breezy style, which doubtless has a therapeutic value for the patients to whom these hints are addressed.

**Diseases of Children.** By H. C. CAMERON, M.A., M.D. (Cantab.), F.R.C.P. (Lond.). Pp. ix., 199. Oxford University Press. 1926. Price 5s.—At the present time the curriculum of a medical student is so crowded that it is impossible to devote an adequate amount of time to the theoretical and practical study of pediatrics. There are numerous excellent books on the subject, but many of these contain an excessive amount of matter which requires prolonged reading to digest. There are, on the other hand, cram books which are quite inadequate and undesirable. Dr. Cameron has succeeded admirably in combining the advantages of both types of text-book and excluding their disadvantages. He has introduced an up-to-date, compact and readable volume on diseases of children which will prove a great boon to medical students. The author has somewhat unusual qualifications for writing a book on pediatrics, for he possesses a wide experience not only as a clinician, but also as an original worker on special branches of the subject, and is thus able to speak with special authority. In this connection the section (pp. 106–109) on " attacks of the abdominal pain, prostration and vomiting " is of special value. We are glad to see that Dr. Cameron has laid special stress on the importance of breast feeding, and failing this, he gives advice as to modifying the best substitute —cow's milk—for the human infant. It is quite cheering to meet with a book that does not encourage the use of proprietary foods, but gives a recipe in one instance for a simple carbohydrate preparation rather than advocating " much advertised preparations which claim a universal application and success." The chapters on " Constipation " and " Inherited Predisposition to Disease " are most instructive, and quite in keeping with the general high standard of the work, which we heartily recommend to all those interested in the subject.

**Obesity.** By Leonard Williams. M.D. (Glas.). 8vo. Pp. 171. Illustrated. Oxford Medical Publications. 1926. Price 10s. 6d.—Dividing his subject into two parts, namely alimentary and endocrine obesity, Dr. Leonard Williams first deals with diagnosis and then with treatment. Throughout the book he writes with a lucidity, a wit and an unconventionality that are rare in a medical work, and which so compel one's attention that the book is difficult to put down. Many of his views will no doubt be challenged, and the chapter entitled " Eve," which deals with obesity in women, is especially likely to excite criticism. The author's views on the necessity for an unfired diet in the treatment of obesity need to be impressed upon the profession, and his simple directions as to the employment of endocrine remedies will be much appreciated at a time when the market is flooded with all sorts of pluriglandular preparations.

**Surgical Operations.** By W. Ibbotson, F.R.C.S. (Edin.). Second edition. Pp. 356. Illustrated. London : Faber and Gwyer. 1926. Price 6s. 6d.—The opening pages of this book contain a description of general surgical technique, the management of an operating theatre and methods of sterilisation. Also in this section is a list of ligature and suture materials, together with an explanation of the methods for rendering them aseptic ; in addition mention is made of various dressings, antiseptics and other surgical necessities. A useful paragraph explains the commoner affixes of operation nomenclature, for example : " ectomy = excision of," etc. Then follows an extensive list of the operations of surgery, with many of their variations and modifications, and the instruments required for each. The whole is arranged in a manner facilitating reference to any particular operation. The final section consists of a series of excellent illustrations of various instruments, named and arranged in alphabetical order. The frontispiece illustrates the Trendelenburg, Fowler and lithotomy positions. Considering the time and trouble which the author must have taken in compiling the work, its uses are extremely limited. It is of most value to a nurse about to undertake the duties of a theatre sister, and might be of assistance to one who contemplated opening a new clinic or to a student about to present himself for an oral examination in surgery.

**Hunter Tod's Diseases of the Ear.** Second Edition. Revised by G. C. Cathcart, M.A. (Edin.), M.D. 8vo. Pp. 333. 4 plates, 87 figures. Oxford Medical Publications. 1926. Price 10s.6d.—A second edition of this excellent little book has been long overdue. An octavo volume just over half an inch thick that gives enough information for the treatment of all but rare ear troubles is certain of a welcome. The book opens with a short chapter on acoustics, which may serve to remind but is too sketchy for reference and might have been omitted. Then follows an excellent account of the anatomy, development, etc., of the ear, which has been re-written to meet modern demands, and of the methods of examination. Disease of the external ear is fully discussed in forty pages. As is natural, disease of the middle ear occupies the bulk of the book. The subject is dealt with clearly and exhaustively: an account of operations on the mastoid sufficient for emergency use is included, and, rather remotely consigned to the last chapter, a short exposition of nasal and pharyngeal conditions affecting the ear. The various intracranial disasters that may complicate otitis media receive the attention they deserve, and a separate chapter is given to the aural complications of acute fevers and other diseases. The description of disorders of the internal ear is disappointing. None of the tests of the vestibule is accurately described—for example, the posterior canals are said to lie in one plane. The old fable that Menière's disease is due to hemorrhage into the labyrinth is repeated. There is one sentence only that might be interpreted as referring to acoustic nerve tumour. Apart from a short account of one method of labyrinthotomy, treatment of internal ear disease, including a note on "the Zund-Burguet electrophonoide," is allotted twenty-one lines. The whole book is generously illustrated. The diagrams and figures are well chosen and reproduced, the directions for various manipulations are clear and concise, there are numerous prescriptions and a complete index. These features, and its general compactness, make this book a very acceptable addition to the library of student or practitioner.

**Treatment of Chronic Deafness by the Electrophonoide.** By G. C. CATHCART, M.A. (Edin.), M.D. 8vo. Pp. 85, one figure. Oxford Medical Publications. 1926. Price 4s. 6d.—The author of this small treatise is manifestly an enthusiastic believer in the method he advocates for the treatment of one of the most intractable of human ills, chronic progressive deafness. Nevertheless, he feels it incumbent on him to rebut a charge of quackery, and to justify his means by the good results, despite an obscure rationale. Briefly, he aims at re-educating the deaf ear, and relates how the apparatus called the Electrophonoide enables him to do this. The description of the device would have been clearer if the instrument illustrated had been of the same pattern as that described. But since we are told only in what order to manipulate the rather complex arrangement of knobs and handles, the internal mechanism being strictly taboo, there is a temptation to regard this machine as on a par with Abram's magic box. Doubtless examination of the actual instrument —it looks costly, if ornamental—would elucidate much. We are confronted with an obviously sincere plea for a new outlook and a new method, and Dr. Cathcart analyses his results in 100 cases, nerve deafness, catarrhal otitis and otosclerosis in equal proportions, treated solely by this apparatus and vibro-massage. In every case, including 6 children under twelve, at least two authorities had pronounced the deafness hopeless and incurable : yet two-thirds of each class found definite benefit. The criterion is the distance at which ordinary speech can be heard. The author has devoted special attention to uniform voice production, and is able to detect after an interval of six or twelve months the loss of hearing measured by a diminution of audibility distance by one foot in thirty ! "However much the patient may improve for the voice, his range of hearing usually remains the same for the tuning-forks." It is unfortunate that twelve "treatments" of each ear are needed before a prognosis can be given : with 32 patients the results of this preliminary course were negative, and treatment was abandoned, representing 768 "treatments." The remaining 68 patients improved. Of these 8 could before treatment hear with either ear at 12 feet or more, another 9 could hear at 16 feet with the better ear, so that in 17 the deafness was mild only. But 26 patients with severe deafness (there were no extreme cases) received a remarkable degree of benefit. Of the whole 68, 15 had three full courses of thirty " treatments " of each ear, 35 had two courses, while 18 needed one course only, a total of 7,980 "treatments," or an average of 117 each. We envy Dr. Cathcart his patience—and his material.

**The Abdomen in Labour.** By R. N. PORRITT, M.R.C.S. Pp. xiv., 76. Illustrated. Oxford Medical Publications. 1926. Price 5s.—In this monograph the author points out the advantages of the abdominal examination of the parturient woman over the vaginal examination. He advocates the dorsal position in labour both for normal and instrumental cases, and in this he will receive the support of a large number of obstetricians. The diagnosis of the commencement and the complications of labour by abdominal examination is fully dealt with and illustrated with simple diagrams, and this chapter is excellent. However, in the next chapter it is very difficult to see how, once the head is in the pelvis, one can determine the progress of labour by abdominal examination alone. The author quite rightly deprecates the use of frequent vaginal examinations, but completely ignores the excellent information which can be obtained by rectal examination without prejudice to the health of the patient. The treatise as a whole is excellent, and if it only stirs up interest and concentrates it in the examination of the abdomen by medical practitioners, it will have achieved a great object and decreased the mortality and morbidity amongst parturient women.

**The Diseases of Women.** Eighth edition. By Sir JOHN BLAND-SUTTON, LL.D., F.R.C.S., and A. E. GILES, M.D., F.R.C.S. (Edin.). Pp. 600. Illustrated. London: Wm. Heinemann Ltd. 1926. Price 15s.—The first edition of this popular book was published in 1897, and the present edition is a store of information. The text has been re-arranged on pathological lines, rather than anatomical. Of particular interest at the present time are the chapters on the uses of radium and X-rays and of endocrine therapy in gynæcology. The general after-results of abdominal operations are recorded and are worthy of study, being based on the observations of 1,000 cases by Dr. Giles. It is noted that memory seems to be affected in about 25 per cent. of cases, and that the deterioration of memory appears to be directly proportioned to the duration of the operation. We welcome this edition very heartily.

**The Quartz Mercury Vapour Lamp.** By J. BELL FERGUSON, M.D. Pp. xii., 106. Illustrated. London: H. K. Lewis & Co. Ltd. 1926. Price 6s.—The busy practitioner of to-day who contemplates adding ultra-violet therapy to his armamentarium will welcome Dr. Ferguson's publication as a really practical and sound guide. This little book contains in concise form a mass of lucid information on the mercury vapour lamp. There is a vast field for the general practitioner in the application of ultra-violet radiation; it is perhaps the very fact that it has been suggested as a panacea that has made many hesitate to install it. No single therapeutic measure can be a "cure all," but the field of beneficial application of ultra-violet radiation is so wide that it should be available in every district. Dr. Ferguson's chapter on its practical use in a modern public health department gives much valuable information as to staff, apparatus and cost. As regards the choice of the source of the radiation, there are many enthusiastic adherents both to the mercury vapour lamp and to the various arc lamps. We cannot agree that the former is alone the ideal type. The greater part of the book, however, is applicable to work with other types. We entirely agree with Dr. Ferguson's statement that actinometers do not help much in deciding the difficult question of dosage. We have found the only reliable method of determining the dose for any patient is that described, exposing small areas of skin to increasing exposures at the first sitting. We agree also that pigmentation is by no means a necessity for, nor a guide to, good results. The author's remarks on cataract are interesting, and his reference to Burge's work significant, so that we feel grateful for his warning as to protection of the eyes of the operator of the lamp. The description of illustrative cases is most instructive and helpful. There are a good bibliography and index. The book is well printed, on good paper, has 38 excellent illustrations, and is, in short, the most helpful work on the subject we have yet met.

**Handbook of Medical Electricity and Radiology.** By J. R. RIDDELL, F.R.F.P.S. Pp. xv., 239. Illustrated. Edinburgh: E. & S. Livingstone. 1926. Price 8s. 6d.—This handbook should prove most useful both to student and to practitioner, who wish to have an account, up to date, concise and on the whole accurate, of those physical agents which are employed in diagnosis and treatment, and which are generally found grouped together in the X-ray and electrical department of a hospital. After three opening chapters on electro-therapeutics and the elementary physics of X-ray technique, the major part of the book is devoted to a clearly-arranged and exceptionally well illustrated group of chapters on X-rays in diagnosis and treatment. Such recent methods as lipoidol bronchoscopy, pyelography, and cholecystography are all considered. There follow two chapters on X-rays and radium in treatment, and the book concludes with brief sections on light therapy and the use of carbon dioxide snow. In spite of a few inaccuracies and several debatable points, this handbook achieves its purpose and can be recommended.

**Invalid Diet.** By Dorothy Morton. Pp. 98. London: Wm. Heinemann Ltd. 1926. Price 5s.—Among the many smaller publications issued for the use of doctors and nurses this book holds a unique place. Few people can think at a moment's notice of a variety of menus suited to special cases of illness, and there is a tendency to repeat one or two classic dishes of "light diet" *ad nauseam.* The young private practitioner equally with the young private nurse will find this book invaluable.

**A Handbook for Nurses.** By J. K. Watson, M.D. 8vo. Pp. xvi., 802. 197 illustrations, 4 coloured plates. London: Faber and Gwyer. 1926. Price 7s. 6d.—The nursing world has always had a very high opinion of this publication. The author seems able to see the nurses' point of view much more clearly than many medical men who write for the instruction of nurses. This new edition is very comprehensive, the anatomy and physiology section is clear and simple, and the diagrams are good; the arrangement of many facts in tabular form is most convenient, and the coloured illustrations of the rashes in infectious fevers are very useful. The lists of instruments—also illustrated—required for different surgical operations are very helpful for junior nurses, and the new chapter on dietetics is a great asset in these days when the patient's diet occupies such an important place in the treatment of both medical and surgical cases.

**First Steps in Nursing.** By E. Margaret Fox, R.R.C., S.R.N. Pp. 140. London: The Scientific Press Ltd. 1924. Price 3s.—*First Steps in Nursing* is a very useful volume for the young probationer to study. It is particularly adapted for the use of probationers in nursing homes, small hospitals and institutions where little practical or theoretical instruction is given during the period of service.

